# Alteration of resting-state network dynamics in autism spectrum disorder based on leading eigenvector dynamics analysis

**DOI:** 10.3389/fnint.2022.922577

**Published:** 2023-01-19

**Authors:** Chaoyan Wang, Lu Yang, Yanan Lin, Caihong Wang, Peichao Tian

**Affiliations:** ^1^Department of Interventional Radiology, The First Affiliated Hospital of Zhengzhou University, Zhengzhou, China; ^2^Department of Pediatrics, The First Affiliated Hospital of Zhengzhou University, Zhengzhou, China

**Keywords:** autism spectrum disorder (ASD), leading eigenvector dynamics analysis (LEiDA), phase-locking (PL) states, frontoparietal control network, occurring probability

## Abstract

**Background:**

Neurobiological models to explain the vulnerability of autism spectrum disorders (ASDs) are scarce, and previous resting-state functional magnetic resonance imaging (rs-fMRI) studies mostly examined static functional connectivity (FC). Given that FC constantly evolves, it is critical to probe FC dynamic differences in ASD patients.

**Methods:**

We characterized recurring phase-locking (PL) states during rest in 45 ASD patients and 47 age- and sex-matched healthy controls (HCs) using Leading Eigenvector Dynamics Analysis (LEiDA) and probed the organization of PL states across different fine grain sizes.

**Results:**

Our results identified five different groups of discrete resting-state functional networks, which can be defined as recurrent PL state overtimes. Specifically, ASD patients showed an increased probability of three PL states, consisting of the visual network (VIS), frontoparietal control network (FPN), default mode network (DMN), and ventral attention network (VAN). Correspondingly, ASD patients also showed a decreased probability of two PL states, consisting of the subcortical network (SUB), somatomotor network (SMN), FPN, and VAN.

**Conclusion:**

Our findings suggested that the temporal reorganization of brain discrete networks was closely linked to sensory to cognitive systems of the brain. Our study provides new insights into the dynamics of brain networks and contributes to a deeper understanding of the neurological mechanisms of ASD.

## 1. Introduction

Autism spectrum disorder (ASD) is a collection of neurodevelopmental disorders marked by a lack of social communication, repetitive behaviors, or interests (American Psychiatric Association, 2013; Saqr et al., 2017; Li et al., 2020). In recent decades, the global prevalence of ASD in children and adolescents has climbed to 0.7–1.5% (Morales-Hidalgo et al., 2018). As a result, a better understanding of the biological origins of many illnesses is a top research focus. Studies of brain network connectivity in ASDs have received much attention in the last decade due to a growing recognition that symptomatology cannot be described just by isolated brain defects (Dinstein et al., 2011; Di Martino et al., 2013, 2017; Müller and Fishman, 2018). Benefiting from the advancement of neuroimaging, extensive evidence has suggested that ASD is linked to abnormal responses in specific brain areas, significant alterations in functional brain networks, and disruptions in neural synchronization among brain areas (Belmonte, 2004; Zhan et al., 2014; Cerliani et al., 2015; Guo et al., 2020). In particular, the underconnectivity hypothesis based on many studies of resting-state functional magnetic resonance imaging (rs-fMRI) proposed that intraregional functional connectivities (FCs) are reduced between the default mode network (DMN) and sensory processing network (Just, 2004; Damarla et al., 2010; Chen et al., 2017; Duan et al., 2017). For example, FCs between the DMN [including the medial prefrontal cortex (mPFC), posterior cingulate cortex (PCC), and precuneus] and the temporal lobe or pallidum gyrus were significantly reduced (Yerys et al., 2015). Other studies of rs-fMRI also showed similar results of reduced FCs between the mPFC and primary motor and sensory cortices (Jung et al., 2019), even the insula temporoparietal junction, and amygdala (von dem Hagen et al., 2012). However, other studies found overconnectivity between brain regions and even a mixture of underconnectivity and overconnectivity in ASD (Keown et al., 2013, 2017; Supekar et al., 2013; Cerliani et al., 2015). Although the underconnectivity or overconnectivity patterns are debatable, FC provides potential new insights to explore the underlying neurological mechanisms for ASD.

The majority of earlier research relied on static functional connectivity (FC) approaches, which assume that FC patterns are consistent across time (Allen et al., 2012; Wang et al., 2020). Theoretical models and empirical evidence, however, show that the dynamic changes in the human brain connectome are linked to continuing rhythmic activity. Recently, researchers have shown more interest in dynamic FC studies and have developed many methods to obtain the profound indicators of dynamic FC (Preti et al., 2017; Du et al., 2018; Wang et al., 2020). Dynamic FC is defined as the time-dependent correlation among brain regions, which has been applied to explore the dynamics of the brain network. Compared to static FC, dynamical FC achieved a better prediction of behavior for the typically developing population (Chen et al., 2017; Li et al., 2020). A recent study also showed that classification based on dynamic connectivity features has significantly higher predictive accuracy in schizophrenia and bipolar disorder (Rashid et al., 2016). Therefore, dynamic FC can capture the underlying dynamic nature of FC alterations and has become a frontier for the exploration of neurological mechanisms in psychiatric disorders. A previous study of dynamic FC found that the mean dwell time of states was significantly different between ASD and developing population groups (Yao et al., 2016). A previous study on the diagnosis prediction of ASD also confirmed that dynamic features, as well as spatiotemporal coherence, can provide more useful information for ASD diagnosis (Wee et al., 2016). In addition, the latest study found that increased variance in dynamic FC was related to ASD symptom severity and suggested that the use of traditional static FC may contribute to the inconsistency of ASD reports (Chen et al., 2017). Nevertheless, it is unclear how dynamics help explain these inconsistent findings.

Although analysis based on the sliding-window approach is most commonly used to probe the alteration of dynamical FC in psychiatric disorders (Sakoğlu et al., 2010), the window size challenges its validity and determines the temporal resolution of dynamic FC patterns (Shine et al., 2015; Hindriks et al., 2016; Deco et al., 2017). Recently, many methodological approaches have been developed to analyze blood oxygenation level-dependent (BOLD) connectivity dynamics of brain activity at a high temporal resolution, such as coactivation patterns (Tagliazucchi et al., 2012; Liu and Duyn, 2013; Karahanoğlu and van de Ville, 2015) or phase coherence patterns (Glerean et al., 2012; Hellyer et al., 2015; Cabral et al., 2017). In particular, the coactivation approaches or their variant forms are only sensitive to simultaneity in the data. Phase coherence techniques can capture temporally delayed relationships and are more sensitive to capturing the ultraslow oscillatory processes governing the formation of resting-state networks (RSNs), which has been confirmed in recent experimental and computational studies (Deco et al., 2009; Ponce-Alvarez et al., 2015; Deco and Kringelbach, 2016; Roberts et al., 2019). Therefore, the best way to characterize dynamic FC remains under debate (Lord et al., 2019). In our study, we used a recently developed data-driven approach named Leading Eigenvector Dynamics Analysis (LEiDA). LEiDA can reduce dimensionality by considering only the relative phase of BOLD signals and capturing the instantaneous phase-locking (PL) patterns (Cabral et al., 2017; Figueroa et al., 2019). The recurrent FC states, also called PL states, can be identified from resting-state time series by operating the LEiDA approach in the temporal domain. The PL states can be characterized by global dynamical statistics (i.e., probabilities of occurrence and duration) and transition profiles on a subject-by-subject level. A previous study indicated that the dynamic properties of recurrent FC states are related to cognitive performance in healthy participants (Cabral et al., 2017). Meanwhile, the PL patterns obtained from the LEiDA approach have shown particular sensitivity to alterations in psychiatric symptoms, such as schizophrenia (Farinha et al., 2022) and major depressive disorders (Figueroa et al., 2019; Alonso Martínez et al., 2020; Wang et al., 2022). However, the recurrent PL patterns identified by LEiDA in ASD have not yet been qualitatively probed. In the current work, we used the LEiDA method to identify recurrent BOLD PL states and even to investigate whether there are specific configurations of PL states that differentiate between ASD and controls.

## 2. Materials and methods

### 2.1. Participants

Resting-state fMRI data in our study were obtained from the NYU Langone Medical Center, which was included in the Autism Brain Imaging Data Exchange (ABIDE) database.^1^ NYU is the largest sample size of individuals who fulfilled the following inclusion criteria: (1) age up to 18 years; (2) predominantly right-handed (Edinburgh Handedness Inventory score >0); (3) estimated full-scale intelligence quotient (IQ) score >80 per the four-subtest Wechsler Abbreviated Scale of Intelligence (WASI), and (4) mean framewise displacement (FD) <0.2 mm. Therefore, a total of 45 individuals with high-functioning ASD and 47 healthy controls (HCs) met the inclusion criteria. The demographic and clinical characteristics of all subjects are shown in Table 1.

**TABLE 1:** Demographics of participants.

	ASD (*n* = 47)	HC (*n* = 45)	Group comparisons (*p*-value)
**Sex (M/F)**	40/7	36/9	0.300^a^
**Age**	11.02 ± 2.28	11.02 ± 2.27	0.987^b^
**Full scale IQ**	107.17 ± 17.34	113.32 ± 14.11	0.072^b^
**ADI-R**			
Social score	18 ± 7.25	−	−
Communication score	15.09 ± 5.16	−	−
RRB score	5.74 ± 3.10	−	−
**ADOS**			
Total score	11.25 ± 4.22	−	−
Social score	9.25 ± 4.13	−	−
Communication score	3.54 ± 1.54	−	−
RRB score	3.25 ± 1.54	−	−

ADI-R, autism diagnostic interview-revised; ADOS, autism diagnostic observation schedule; RRB, restricted and repetitive behaviors.

^a^Chi-square test.

^b^Two-sample *t*-test.

### 2.2. Image acquisition and pre-processing

Magnetic resonance imaging data of all subjects were acquired using a 3 Tesla Siemens Allegra scanner. Details regarding acquisition parameters are provided on the ABIDE website.^2^ In brief, rs-fMRI data were acquired using an echo planner imaging (EPI) sequence sensitive to BOLD contrast with the scan parameters repetition time (TR) = 2000 ms, echo time (TE) = 33 ms, flip angle (FA) = 90°, matrix = 30 × 80 × 80, and voxel size = 3 mm × 3 mm × 4 mm, and each scanning session lasted for 6 min. In addition, a high-resolution three-dimensional T1-weighted image was also scanned for anatomic reference.

We adopted the standard pipeline for pre-processing of fMRI data using the Data Processing and Analysis for Brain Imaging toolbox (DPABI)^3^ (Yan et al., 2016). The pre-processing steps were performed as described in a previous study (Chen et al., 2017). In brief, the main steps included the following: (1) removing the first 10 time points; (2) slice timing correction; (3) head motion correction, importantly, subjects with high levels of head motion were excluded (maximum motion >2 mm or 2° rotation or mean FD >0.2 mm) (Power et al., 2012; Yang et al., 2014); (4) regressing out nuisance covariates, which included signals from white matter (WM) and cerebrospinal fluid (CSF), as well as 24 rigid body motion parameters; (5) normalization to Montreal Neurological Institute (MNI) standard space at 3-mm isotropic voxel resolution by diffeomorphic Anatomical Registration Through Exponentiated Lie Algebra (DARTEL); (6) spatial smoothing with a Gaussian kernel (full width at half maximum (FWHM) = 6 mm); (7) bandpass filtering (0.01–0.08 Hz); and (8) extracting the averaged rs-fMRI time courses in 90 brain regions based on the automated anatomical labeling (AAL) template.

### 2.3. Leading eigenvector dynamics analysis (LEiDA)

**FIGURE 1:**
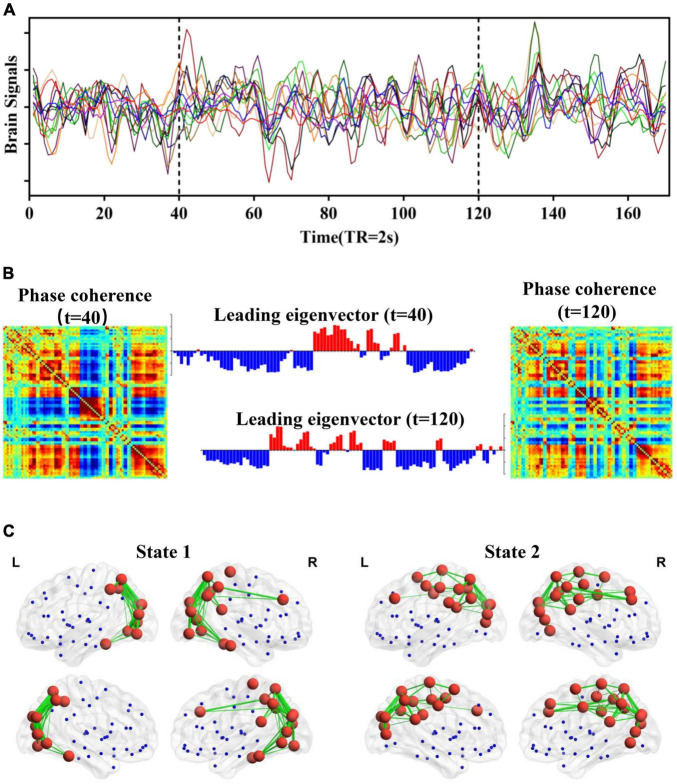
Schematic illustration of LEiDA. **(A)** BOLD signals based on the AAL template. **(B)** The phase coherence of BOLD signals and the corresponding leading eigenvector at *t* = 40 and 120 (TR). **(C)** A network in cortical space for each PL state.

In our study, a novel framework called LEiDA was adopted to identify the BOLD PL states as a stochastic subdivision of regular and persistent brain states (Cabral et al., 2017). LEiDA could calculate the leading eigenvector of the BOLD phase-coherence matrices over time to capture the connectivity patterns, which is a data-driven method. Previous evidence has suggested that the LEiDA framework is highly flexible, robust, and precise (Glerean et al., 2012; Ponce-Alvarez et al., 2015; Cabral et al., 2017), allowing for recurrent states that were detected and characterized in resting state and task conditions in the healthy brain. It can also distinguish the abnormal brain states in psychiatric diseases, such as schizophrenia (Farinha et al., 2022), major depressive disorders (Figueroa et al., 2019; Alonso Martínez et al., 2020; Wang et al., 2022), and trait self-reflectiveness (Larabi et al., 2020), and the alteration of brain states in psilocybin (Lord et al., 2019) and sleep (Deco et al., 2019). The fundamental framework of LEiDA is shown in Figure 1, and the detailed steps are mentioned later.

First, the PL matrix at each time point was calculated to capture the amount of interregional BOLD signal synchrony at any given time point for each subject (including 45 ASD and 47 HCs). Specifically, the BOLD time series of each brain region was subsequently Hilbert-transformed to yield the phase evolution of the BOLD time course of this brain region. The phase coherence between each pair of brain regions at a given time was then estimated through the cosine of the phase difference using Equation (1):


(1) dPL(i,j,t)=cos(θ(i,t)−θ(j,t))

where θ(*i*,*t*) and θ(*j*,*t*) are the time-varying phases of the BOLD signal in the *i* region and *j*region at a given time *t* and are computed using the Hibert transform of the BOLD regional timecourse. Based on the cosine of the phase difference, two brain regions with temporarily aligned BOLD signals (i.e., with a similar phase) at a given time point will have a PL value close to 1, two regions with orthogonally developing BOLD signals will have zero PL values, and two regions with 180° phase differences will have PL values equal to −1. The PL matrix at each time point is undirected and symmetric, with values ranging between −1 and 1. Then, the leading eigenvector of the PL matrix at each time point was calculated to reduce the dimensionality of the PL matrix. The leading eigenvector captures the main orientation of BOLD phases over all brain regions, and each value of the leading eigenvector represents the projection of the BOLD phase in each brain region into the leading eigenvector. The sign of elements can be used to divide the brain area into two communities, and the magnitude of elements indicates the contribution of their communities. Upon computing the leading eigenvector of the PL matrix for each time point of each subject, we used the k-means clustering algorithm to identify the recurrent PL patterns for ASD and HCs. Notably, we aimed to explore whether there are specific and robust PL states with abnormal dynamical characteristics for ASD. The number of clusters (k) is a free parameter, and a higher k reveals a rare and more fine-grained network configuration. The optimal number of PL states is not a consensus. Therefore, to verify the robustness of the abnormal PL state configuration in our study, the number of clusters varies over a wide range from 3 to 20. For each k, how each PL state was significantly altered between ASDs and HCs was examined.

### 2.4. Statistical analysis

Based on the PL states identified by LEiDA, the probability of occurrence of each PL state for each subject and each k was assessed. In particular, the probability of occurrence, also called fractional occupancy, is the ratio of the number of epochs assigned to a given PL state divided by the total number of epochs (TRs). Then, the occurrence probability of each PL state was compared between the subjects with ASD and HCs using non-parametric permutation-based *t*-tests with 5000 permutations and Bonferroni’s correction. Finally, to evaluate the consistency of PL states with significantly alternated fractional occupancy, we calculated Pearson’s correlation between centroid vectors of PL states.

## 3. Results

### 3.1. Significant alteration of PL states identified in ASD

**FIGURE 2:**
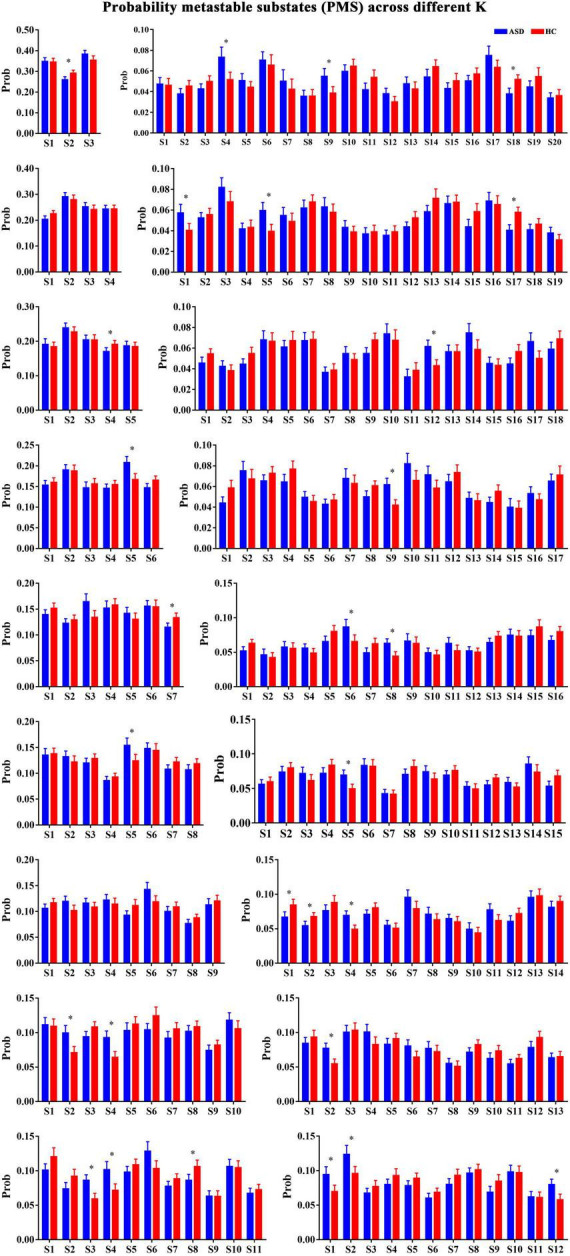
The probabilities of PL states across *k* = 3–20. *Represented the significant difference between ASD and HC.

The repertoire of the PL state identified from the BOLD that time series depends on the number of clusters. In general, a higher number of clusters can result in more fine-grained and less frequent brain networks. Importantly, the main purpose of our study was not to determine the optimal number of FC states but instead to probe the robust FC configurations that significantly and consistently differentiate ASD from HCs. Therefore, we calculated and compared the occurrence probability of each FC state for each clustering model in patients with ASD and HCs. As shown in Figure 2, we found that of the 18-partition model considered (with k ranging from 3 to 20), 16 solutions revealed the identified PL states that occurred during the temporal organization in subjects with ASD (Figure 2). Specifically, compared to controls, the occurrence probabilities of the PL state Sk (*k* = 3 to 18) were significantly decreased in subjects with ASD from *k* = 3 (S2: *p* = 0.007), 5 (S4: *p* = 0.001), 11 (S8: *p* = 4.27 × 10^–3^), 14 (S1: *p* = 3.57 × 10^–3^; S2: *p* = 3.35 × 10^–3^), 19 (S17: *p* = 2.63 × 10^–4^), and 20 (S18: *p* = 5.51 × 10^–4^). Correspondingly, our study also identified PL states with increased occurrence probabilities for *k* = 6 (S5: *p* = 3.3 × 10^–3^), 7 (S7: *p* = 4.57 × 10^–3^), 8 (S5: *p* = 6.25 × 10^–3^), 10 (S2: *p* = 1.5 × 10^–3^; S4: *p* = 6.01 × 10^–4^), 11 (S3: *p* = 8.18 × 10^–4^; S4: *p* = 1.45 × 10^–3^), 12 (S1: *p* = 3.25 × 10^–3^; S2: *p* = 3.92 × 10^–3^; S1: *p* = 1.92 × 10^–3^), 13 (S2: *p* = 3.08 × 10^–4^), 14 (S4: *p* = 5.11 × 10^–4^), 15 (S5: *p* = 8.64 × 10^–4^, 16 (S6: *p* = 3.13 × 10^–3^; S6: *p* = 6.25 × 10^–4^), 17 (S9: *p* = 5.71 × 10^–3^), 18 (S12: *p* = 4.44 × 10^–3^), 19 (S1: *p* = 2.11 × 10^–4^; S1: *p* = 1.26 × 10^–3^), and 20 (S4: *p* = 1.23 × 10^–3^; S4: *p* = 2.15). All between-group comparisons were performed using a non-parametric permutation *t*-test and Bonferroni’s correction.

### 3.2. Spatial activation map of the alteration states

**FIGURE 3:**
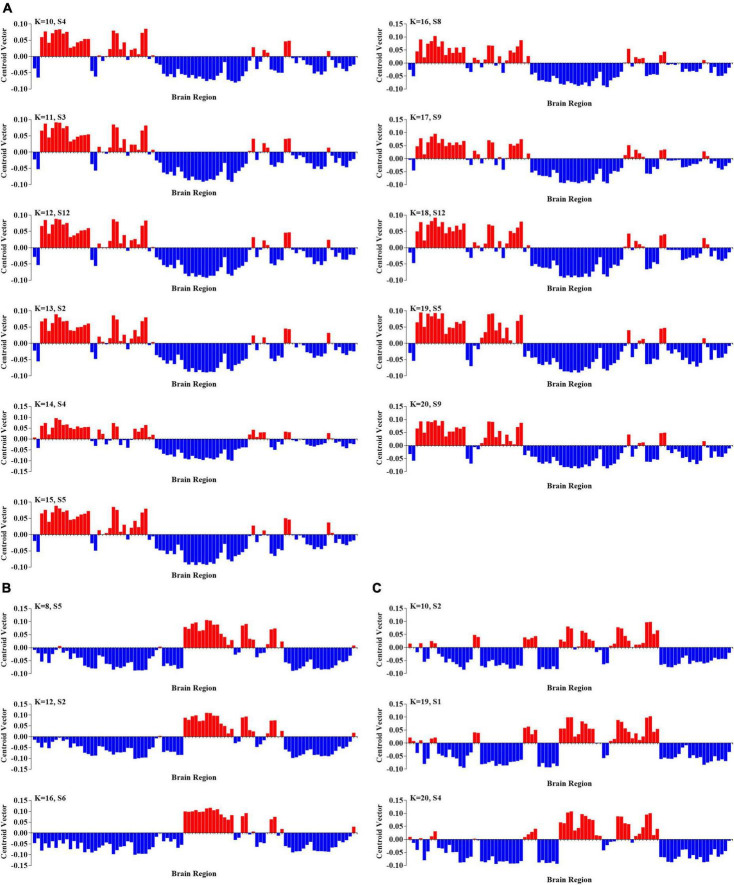
Three groups of PL states with significantly increasing probabilities in ASD. **(A)** The PL states in group I at *k* = 10–20. **(B)** The PL states in group II at *k* = 8, 12, and 16; and **(C)** the PL states in group III at *k* = 10, 19, and 20.

**FIGURE 4:**
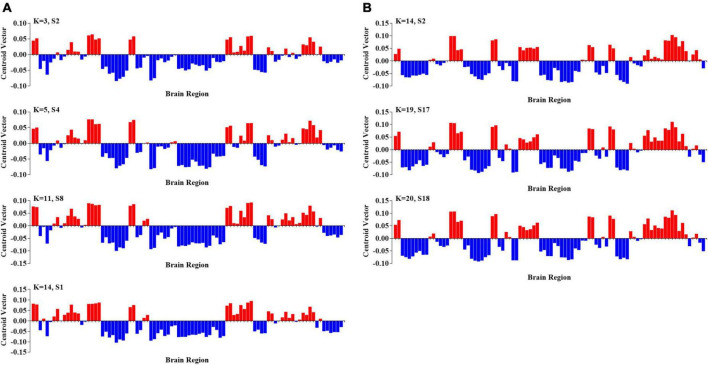
Two groups of PL states with significantly increasing probabilities in ASD. **(A)** The PL states in group IV at *k* = 3, 5, 11, and 14; and **(B)** the PL states in group V at *k* = 2, 17, and 18.

To further probe the spatial activation map of the significantly altered PL states in ASD, we calculated the Pearson’s correlation coefficient between each pair of characterizations (vector of clustering centers) of the significantly altered PL states across *k* = 3–20. In our study, we found that the PL states could be divided into five groups of states and that PL states in the same group had higher similarity (Pearson’s *r* > 0.9 for all paired FC states in one group). In particular, as shown in Figure 3, the PL states whose occurrence probabilities were significantly increased in ASD were organized into three groups. The PL states in group I were identified from k ranging from 10 to 20, which indicated that these states referred to variant forms of the same underlying PL states in more detailed partitions (*K* > 10). The spatial activation map of the PL state in group I was characterized by the insula, caudate, angular gyrus, frontal gyrus, anterior and middle cingulate cortex (ACC and MCC), and inferior parietal gyrus (Figure 3A). The PL states from *k* = 8 (S5), 12 (S2), and 16 (S6) were divided into group II and from *K* = 10 (S2), 19 (S1), and 20 (S4) into group III. The PL states from group II were characterized by the angular, precuneus, cuneus, occipital gyrus, superior parietal gyrus, and fusiform gyrus (Figure 3B), and those from group III were characterized by the middle frontal gyrus, the middle and post cingulate cortex (MCC and PCC), the occipital gyrus, the central lobe, the angular, inferior, and superior parietal gyrus, and the precuneus and cuneus (Figure 3C). Correspondingly, as shown in Figure 4, the PL states with significantly decreased occurrence probabilities in ASD were organized into two groups of states. One (group IV) consisted of the PL states identified from *K* = 3 (S2), 5 (S4), 11 (S8), and 14 (S1), and another (group V) consisted of the PL states from 14 (S2), 19 (S17), and 20 (S18). PL states in the former group were characterized by the subcortical cortex (including insula, caudate, putamen, and thalamus), the middle inferior frontal gyrus and orbitofrontal, the central gyrus, the supplementary motor area, the paracentral lobule, the parietal gyrus, the superior temporal gyrus, the temporal pole, and the MCC (Figure 4A). PL states in the latter group were characterized by the subcortical cortex (insula, amygdala, putamen, hippocampus and parahippocampus, and pallidum gyrus), the middle and superior temporal gyrus, the temporal pole, and the central gyrus (Figure 4B).

### 3.3. Comparison between PL states and resting-state networks

**FIGURE 5:**
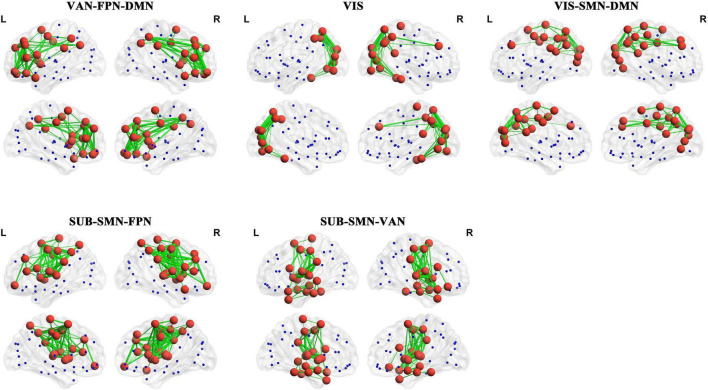
The corresponding RSNs of the PL states. The red nodes represent that the corresponding elements of centroid vectors are positive and the blue nodes represent that the corresponding elements are negative. The details are shown in Supplementary Table 2.

Finally, we computed the spatial Pearson correlation between the canonical resting-state networks (RSNs) and each PL state. In particular, consistent with previous studies (Thomas Yeo et al., 2011; Lord et al., 2019), we first obtained the contribution of each AAL brain area to 7 RSNs by calculating the number of voxels of each brain area in each RSN. The contribution of each AAL brain area to 7 RSNs was show in Supplementary Table 1. Then, we computed the bivariate correlation between centroid vectors of each PL state and the contribution of each AAL brain area to 7 RSNs. Our results showed striking spatial similarities between 7 RSNs with PL states with a significantly altered occurrence probability in ASD. Specifically, as shown in Figure 5, PL states from group I were called the “VAN-FPN-DMN state,” including the ventral attention network (VAN: insula, MCC, and supramarginal gyrus, *r* = 0.37, *p* = 1.08 × 10^–4^), frontoparietal control network (FPN: the middle and orbital frontal gyrus, the opercular and triangular part of the inferior frontal gyrus and the inferior parietal gyrus, *r* = 0.643, *p* = 8.21 × 10^–12^), and (DMN: superior frontal gyrus, the orbital and inferior frontal gyrus, the superior and medial frontal gyrus, ACC, and angular gyrus, *r* = 0.36, *p* = 5.09 × 10^–4^). PL states from group II were called “VIS states,” including the visual network (VIS: occipital gyrus, fusiform gyrus, lingual gyrus, calcarine area, and cuneus, *r* = 0.72, *p* = 2.05 × 10^–15^). PL states from group III were indicated as the “VIS-SMN-DMN state,” including VIS (the superior and middle occipital gyrus, calcarine area, and cuneus, *r* = 0.50, *p* = 4.95 × 10^–7^), somatomotor network (SMN: postcentral gyrus and paracentral lobule, *r* = 0.38, *p* = 8.45 × 10^–6^), and DMN (precuneus, angular, superior frontal gyrus, and PCC, *r* = 0.38, *p* = 8.45 × 10^–6^). PL states from group IV consisted of an extensive network and were called the “SUB-SMN-FPN state,” including the subcortical network (SUB: caudate, putamen, and pallidum), SMN (precentral gyrus, postcentral gyrus, Rolandic operculum, paracentral lobule, Heschl’s gyrus, and superior temporal gyrus *r* = 0.55, *p* = 1.16 × 10^–18^), and FPN (inferior and middle frontal gyrus and inferior parietal gyrus, *r* = 0.58, *p* = 1.73 × 10^–9^). PL states from group V were indicated as the “SUB-SMN-VAN state,” which consists of SUB (hippocampus, amygdala, putamen, thalamus, and pallidum), SMN (precentral gyrus, postcentral gyrus, Rolandic operculum, paracentral lobule, Heschl’s gyrus, and superior temporal gyrus, *r* = 0.36, *p* = 4.71 × 10^–4^), and VAN (VAN: insula and supramarginal gyrus, *r* = 0.58, *p* = 2.96 × 10^–9^).

## 4. Discussion

Our study probed the dynamics of brain networks reoccurring over time during the resting state for ASD. Specifically, we identified five different groups of discrete resting-state functional networks, which can be defined as recurrent BOLD PL patterns over time (PL states). Meanwhile, we also found that these PL states were characterized by significantly increased or decreased occurrence probability for ASD and were closely linked to sensory-to-cognitive systems of the brain. Our study provides new insights into the dynamics of brain networks and contributes to a deeper understanding of the neurological mechanisms of ASD.

Most previous studies of FC in ASD relied on the assumption that connectivity patterns remain constant over time (Cerliani et al., 2015; Chen et al., 2017; Di Martino et al., 2017). However, the whole-brain functional network changes dynamically with ongoing rhythmic activity (Allen et al., 2012; Cavanna et al., 2018; Harlalka et al., 2019). This variability in the whole-brain functional network can be tracked in different brain states by using dynamic FC. Knowledge of the dynamic patterns across brain regions may allow for a better understanding of resting-state functional organization and processing. Numerous studies have indicated that dynamic FC analytical techniques are a promising avenue of research in ASD and can broaden our understanding of changes in functional brain organization among individuals with the disorder (He et al., 2018; Rashid et al., 2018; Harlalka et al., 2019; Mash et al., 2019; Li et al., 2020). However, the best way to characterize dynamic FC remains under debate. In our study, we adopted the LEiDA method to characterize FC at the instantaneous level and found a special and robust PL state configuration. Examining partition modes from *k* = 3 to 20 clusters, our results found special and robust PL states, whose occurrence probabilities were significantly altered in patients with ASD compared to HCs. Specifically, there are three types of PL states that occurred more in ASD, and the other two types of PL states that occurred less in ASD across models with k ranging from 3 to 20. Our results are consistent with previous findings that patients with ASD have special patterns of brain PL state organization that are significantly different from those of HCs. Previous studies found that there were significantly different occurrences in multiple functional states between patients with ASD and HCs. For example, subjects with ASD spent more time in a state characterized by weak dynamic FC patterns and less negative dynamic FC between the DMN and other sensory networks, while healthy individuals spent more time in a state with both positive and negative dynamic FC patterns (Rabany et al., 2019). In addition, recent work based on the hidden Markov Model (HMM) found that of the 19 HMM states, four HMM states have significantly higher fractional occupancies and the other four states have fewer fractional occupancies in ASD (Lin et al., 2022). Therefore, our results indicated the altered pattern of brain connectivity recurrently and consistently in ASD, which is robust across a range of partition models.

In our study, the identified PL states across k ranging from 3 to 20 could be divided into five groups of states, and PL states in the same group had higher similarity (Pearson’s *r* > 0.9 for all paired FC states in one group). Five types of PL states were closely linked to sensory-to-cognitive systems in the brain. The occurrence probabilities of three of five groups of PL states were significantly increased in ASD. Three PL states were defined as the “VAN-FPN-DMN state,” “VIS state,” and “VIS-SMN-DMN state.” In particular, abnormal dynamics of the “VAN-FPN-DMN state” were robustly identified across a large range of k-means solutions. This state consists of an extensive network connecting frontal areas—important for cognitive control—with the DMN (superior and medial frontal gyrus, ACC, and angular) and VAN (including insula and MCC). Our results are consistent with previous studies reporting overconnectivity between these RSNs.

For example, a recent study used sliding window analysis to probe the alteration in transient connectivity in ASD and found that an overall trend toward overconnectivity is largely driven by the network-level trend toward overconnectivity among DMN, FPN, and VAN. The significantly altered overconnectivity between the DMN and FPN regions was robust across the different numbers of clusters (Mash et al., 2019). Moreover, other PL states found to be more idiosyncratic in ASD were the “VIS state” and “VIS-SMN-DMN state,” which are mainly characterized by the VIS (including occipital gyrus, fusiform gyrus, lingual gyrus, calcarine area, and cuneus) with DMN (including the precuneus, angular, superior frontal gyrus, and PCC). A recent study found that aberrant FC between DMN and primary visual cortex (PVC) as well as visual association circuitry in occipitotemporal cortex social is closely related to visual engagement difficulties, which are central early developmental features of ASD (Lombardo et al., 2019). Together with our results, these findings consistently highlight the potential role of the neural circuits associated with the sensory and cognitive networks (including VIS, DMN, FPN, and VAN) in the pathological mechanisms of ASD.

Furthermore, we also found that the occurrence probabilities of the other two PL states were significantly decreased in ASD. These two states were mainly characterized by SUBs (including the hippocampus, amygdala, caudate, thalamus, putamen, and pallidum), SMN (including the pre- and postcentral gyrus, paracentral lobule, and superior temporal gyrus), FPN, and VAN. The common features of ASD are abnormal sensory and motor behaviors, such as clumsiness, hyper and hypo-sensitivities, and motor coordination impairments (Whyatt and Craig, 2013). Previous static FC studies in ASD have demonstrated impaired functional synchronization between visual networks and sensory-motor networks (Nebel et al., 2016; Oldehinkel et al., 2019). Interhemispheric FC was significantly decreased in the sensorimotor cortex in individuals with ASD (Anderson et al., 2011). In addition, a recent study based on static functional hub distribution showed significantly reduced FC in the pre- and postcentral gyrus in ASD (Lee et al., 2016). Meanwhile, an increasing number of clinical and experimental studies have reported atypical sensory processing in ASD, which is qualified as hypo-reactivity or hyperreactivity to sensory stimuli and could enhance sensory-perceptual processing and discrimination (Baron-Cohen et al., 2009; Marco et al., 2011; Cerliani et al., 2015). Consistent with these findings, the configuration reduction of brain states with SMN and SUBs over time observed in the current study further emphasized abnormal dynamic functional coordination of the sensorimotor network in the autistic brain and may contribute to the aberrant sensory and motor processing in ASD.

In summary, the present study used a novel and data-driven LEiDA approach to examine the instantaneous dynamics of brain activities and found five different and robust groups of discrete resting-state functional networks, and their occurrence probabilities were significantly altered in ASD. Our findings provide new insights into aberrations in dynamic brain network connectivity in ASD and contribute to a deeper understanding of the neurological mechanisms of ASD.

## Data availability statement

Publicly available datasets were analyzed in this study. This data can be found here: Autism Brain Imaging Data Exchange (ABIDE), http://fcon_1000.projects.nitrc.org/indi/abide/.

## Ethics statement

The studies involving human participants were reviewed and approved by the Ethics Committee of Zhengzhou University. Written informed consent to participate in this study was provided by the participants’ legal guardian/next of kin.

## Author contributions

ChW and PT contributed to the design of the study. LY, YL, and CaW performed the statistical analysis and wrote the first draft of the manuscript. All authors contributed to manuscript revision and read and approved the submitted version.

## Footnotes

1
http://fcon_1000.projects.nitrc.org/indi/abide/

2
http://fcon_1000.projects.nitrc.org/indi/abide/scan_params/NYU/

3
http://rfmri.org/dpabi
