# Supplementary material for: Alteration of resting-state network dynamics in autism spectrum disorder based on leading eigenvector dynamics analysis
Source: Front Integr Neurosci. 2023 Jan 19;16:922577. doi: 10.3389/fnint.2022.922577 (PMC9892631; doi:10.3389/fnint.2022.922577)
Supplement: Supplementary file 1 [file Table_1.docx]

Table S1 The contribution of each AAL brain area to 7 RSNs defined by Yeo et al.(2011)

| **Index** | **Brain area** | **x** | **y** | **z** | **VIS** | **SMN** | **DAN** | **VAN** | **Limbic** | **FPN** | **DMN** |
| --- | --- | --- | --- | --- | --- | --- | --- | --- | --- | --- | --- |
| 1 | PreCG.L | -38.65 | -5.68 | 50.94 | 0 | 9313 | 6324 | 864 | 0 | 2818 | 1040 |
| 2 | PreCG.R | 41.37 | -8.21 | 52.09 | 0 | 11271 | 4253 | 1957 | 0 | 2048 | 0 |
| 3 | SFGdor.L | -18.45 | 34.81 | 42.2 | 0 | 475 | 2545 | 1274 | 0 | 2650 | 12468 |
| 4 | SFGdor.R | 21.9 | 31.12 | 43.82 | 0 | 2275 | 2910 | 1354 | 5 | 5411 | 10500 |
| 5 | ORBsup.L | -16.56 | 47.32 | -13.31 | 0 | 0 | 0 | 5 | 4186 | 532 | 1524 |
| 6 | ORBsup.R | 18.49 | 48.1 | -14.02 | 0 | 0 | 0 | 0 | 4378 | 1871 | 172 |
| 7 | MFG.L | -33.43 | 32.73 | 35.46 | 0 | 1 | 1336 | 4426 | 0 | 13600 | 9390 |
| 8 | MFG.R | 37.59 | 33.06 | 34.04 | 0 | 51 | 1203 | 3390 | 0 | 24957 | 2947 |
| 9 | ORBmid.L | -30.65 | 50.43 | -9.62 | 0 | 0 | 0 | 58 | 1418 | 3171 | 1169 |
| 10 | ORBmid.R | 33.18 | 52.59 | -10.73 | 0 | 0 | 0 | 0 | 1338 | 5430 | 130 |
| 11 | IFGoperc.L | -48.43 | 12.73 | 19.02 | 0 | 14 | 385 | 1474 | 0 | 2694 | 1604 |
| 12 | IFGoperc.R | 50.2 | 14.98 | 21.41 | 0 | 14 | 1897 | 1897 | 0 | 4318 | 314 |
| 13 | IFGtriang.L | -45.58 | 29.91 | 13.99 | 0 | 0 | 2 | 814 | 0 | 9449 | 4499 |
| 14 | IFGtriang.R | 50.33 | 30.16 | 14.17 | 0 | 0 | 139 | 1659 | 0 | 7938 | 1810 |
| 15 | ORBinf.L | -35.98 | 30.71 | -12.11 | 0 | 0 | 0 | 167 | 2646 | 1175 | 7487 |
| 16 | ORBinf.R | 41.22 | 32.23 | -11.91 | 0 | 0 | 0 | 424 | 2020 | 2755 | 5319 |
| 17 | ROL.L | -47.16 | -8.48 | 13.95 | 0 | 4089 | 13 | 2111 | 0 | 72 | 0 |
| 18 | ROL.R | 52.65 | -6.25 | 14.63 | 0 | 5330 | 102 | 2886 | 0 | 0 | 0 |
| 19 | SMA.L | -5.32 | 4.85 | 61.38 | 0 | 3591 | 257 | 4518 | 0 | 1546 | 2795 |
| 20 | SMA.R | 8.62 | 0.17 | 61.85 | 0 | 5753 | 0 | 5421 | 0 | 1011 | 1137 |
| 21 | OLF.L | -8.06 | 15.05 | -11.46 | 0 | 0 | 0 | 0 | 895 | 0 | 64 |
| 22 | OLF.R | 10.43 | 15.91 | -11.26 | 0 | 0 | 0 | 0 | 1047 | 0 | 80 |
| 23 | SFGmed.L | -4.8 | 49.17 | 30.89 | 0 | 0 | 0 | 135 | 0 | 1962 | 14286 |
| 24 | SFGmed.R | 9.1 | 50.84 | 30.22 | 0 | 0 | 0 | 34 | 2 | 1622 | 11148 |
| 25 | ORBsupmed.L | -5.17 | 54.06 | -7.4 | 0 | 0 | 0 | 0 | 914 | 0 | 3661 |
| 26 | ORBsupmed.R | 8.16 | 51.67 | -7.13 | 0 | 0 | 0 | 0 | 1172 | 2 | 4684 |
| 27 | REC.L | -5.08 | 37.07 | -18.14 | 0 | 0 | 0 | 0 | 5025 | 0 | 717 |
| 28 | REC.R | 8.35 | 35.64 | -18.04 | 0 | 0 | 0 | 0 | 3875 | 0 | 1038 |
| 29 | INS.L | -35.13 | 6.65 | 3.44 | 0 | 1779 | 0 | 8232 | 93 | 1248 | 928 |
| 30 | INS.R | 39.02 | 6.25 | 2.08 | 0 | 2708 | 0 | 7803 | 63 | 951 | 939 |
| 31 | ACG.L | -4.04 | 35.4 | 13.95 | 0 | 0 | 0 | 1688 | 96 | 1302 | 6920 |
| 32 | ACG.R | 8.46 | 37.01 | 15.84 | 0 | 0 | 0 | 1262 | 0 | 1826 | 6339 |
| 33 | DCG.L | -5.48 | -14.92 | 41.57 | 0 | 3709 | 5 | 5952 | 0 | 723 | 3218 |
| 34 | DCG.R | 8.02 | -8.83 | 39.79 | 0 | 3213 | 37 | 7285 | 0 | 2641 | 2532 |
| 35 | PCG.L | -4.85 | -42.92 | 24.67 | 0 | 0 | 0 | 0 | 0 | 73 | 2479 |
| 36 | PCG.R | 7.44 | -41.81 | 21.87 | 0 | 0 | 0 | 0 | 0 | 107 | 1264 |
| 37 | HIP.L | -25.03 | -20.74 | -10.13 | 0 | 0 | 0 | 0 | 0 | 0 | 25 |
| 38 | HIP.R | 29.23 | -19.78 | -10.33 | 3 | 0 | 0 | 0 | 3 | 0 | 0 |
| 39 | PHG.L | -21.17 | -15.95 | -20.7 | 636 | 0 | 12 | 0 | 2002 | 0 | 756 |
| 40 | PHG.R | 25.38 | -15.15 | -20.47 | 1409 | 0 | 0 | 0 | 2664 | 0 | 316 |
| 41 | AMYG.L | -23.27 | -0.67 | -17.14 | 0 | 0 | 0 | 0 | 159 | 0 | 0 |
| 42 | AMYG.R | 27.32 | 0.64 | -17.5 | 0 | 0 | 0 | 0 | 100 | 0 | 0 |
| 43 | CAL.L | -7.14 | -78.67 | 6.44 | 13759 | 0 | 0 | 0 | 0 | 0 | 1765 |
| 44 | CAL.R | 15.99 | -73.15 | 9.4 | 10156 | 0 | 0 | 0 | 0 | 0 | 930 |
| 45 | CUN.L | -5.93 | -80.13 | 27.22 | 7971 | 0 | 30 | 0 | 0 | 482 | 1708 |
| 46 | CUN.R | 13.51 | -79.36 | 28.23 | 8565 | 0 | 129 | 0 | 0 | 935 | 419 |
| 47 | LING.L | -14.62 | -67.56 | -4.63 | 13888 | 0 | 0 | 0 | 0 | 0 | 158 |
| 48 | LING.R | 16.29 | -66.93 | -3.87 | 14401 | 0 | 0 | 0 | 0 | 0 | 288 |
| 49 | SOG.L | -16.54 | -84.26 | 28.17 | 7224 | 0 | 1029 | 0 | 0 | 98 | 55 |
| 50 | SOG.R | 24.29 | -80.85 | 30.59 | 6737 | 0 | 1447 | 0 | 0 | 316 | 0 |
| 51 | MOG.L | -32.39 | -80.73 | 16.11 | 14736 | 0 | 4016 | 0 | 0 | 421 | 2106 |
| 52 | MOG.R | 37.39 | -79.7 | 19.42 | 10579 | 0 | 3428 | 0 | 0 | 324 | 505 |
| 53 | IOG.L | -36.36 | -78.29 | -7.84 | 4190 | 0 | 1188 | 0 | 0 | 20 | 0 |
| 54 | IOG.R | 38.16 | -81.99 | -7.61 | 6383 | 0 | 3 | 0 | 0 | 0 | 0 |
| 55 | FFG.L | -31.16 | -40.3 | -20.23 | 8379 | 0 | 2191 | 0 | 4726 | 0 | 717 |
| 56 | FFG.R | 33.97 | -39.1 | -20.18 | 11855 | 0 | 490 | 0 | 5518 | 0 | 104 |
| 57 | PoCG.L | -42.46 | -22.63 | 48.92 | 0 | 21930 | 1189 | 850 | 0 | 0 | 0 |
| 58 | PoCG.R | 41.43 | -25.49 | 52.55 | 0 | 19658 | 3439 | 253 | 0 | 0 | 0 |
| 59 | SPG.L | -23.45 | -59.56 | 58.96 | 220 | 2499 | 9380 | 0 | 0 | 1052 | 128 |
| 60 | SPG.R | 26.11 | -59.18 | 62.06 | 404 | 614 | 12538 | 102 | 0 | 405 | 0 |
| 61 | IPL.L | -42.8 | -45.82 | 46.74 | 3 | 866 | 7499 | 314 | 0 | 7509 | 1976 |
| 62 | IPL.R | 46.46 | -46.29 | 49.54 | 25 | 40 | 3626 | 1 | 0 | 5787 | 958 |
| 63 | SMG.L | -55.79 | -33.64 | 30.45 | 0 | 380 | 1107 | 6004 | 0 | 124 | 827 |
| 64 | SMG.R | 57.61 | -31.5 | 34.48 | 0 | 255 | 2404 | 6716 | 0 | 3436 | 978 |
| 65 | ANG.L | -44.14 | -60.82 | 35.59 | 0 | 0 | 20 | 0 | 0 | 702 | 7258 |
| 66 | ANG.R | 45.51 | -59.98 | 38.63 | 0 | 0 | 1568 | 0 | 0 | 2988 | 7387 |
| 67 | PCUN.L | -7.24 | -56.07 | 48.01 | 238 | 2934 | 6028 | 1942 | 0 | 2467 | 8302 |
| 68 | PCUN.R | 9.98 | -56.05 | 43.77 | 618 | 550 | 7090 | 1346 | 0 | 2975 | 7036 |
| 69 | PCL.L | -7.63 | -25.36 | 70.07 | 0 | 6717 | 2 | 4 | 0 | 0 | 0 |
| 70 | PCL.R | 7.48 | -31.59 | 68.09 | 0 | 3799 | 1 | 367 | 0 | 0 | 0 |
| 71 | CAU.L | -11.46 | 11 | 9.24 | 0 | 0 | 0 | 0 | 69 | 0 | 0 |
| 72 | CAU.R | 14.84 | 12.07 | 9.42 | 0 | 0 | 0 | 0 | 30 | 0 | 0 |
| 73 | PUT.L | -23.91 | 3.86 | 2.4 | 0 | 0 | 0 | 37 | 0 | 0 | 0 |
| 74 | PUT.R | 27.78 | 4.91 | 2.46 | 0 | 0 | 0 | 94 | 0 | 0 | 0 |
| 75 | PAL.L | -17.75 | -0.03 | 0.21 | 0 | 0 | 0 | 0 | 0 | 0 | 0 |
| 76 | PAL.R | 21.2 | 0.18 | 0.23 | 0 | 0 | 0 | 0 | 0 | 0 | 0 |
| 77 | THA.L | -10.85 | -17.56 | 7.98 | 0 | 0 | 0 | 0 | 0 | 0 | 0 |
| 78 | THA.R | 13 | -17.55 | 8.09 | 0 | 0 | 0 | 0 | 0 | 0 | 0 |
| 79 | HES.L | -41.99 | -18.88 | 9.98 | 0 | 1767 | 0 | 27 | 0 | 0 | 0 |
| 80 | HES.R | 45.86 | -17.15 | 10.41 | 0 | 1877 | 0 | 8 | 0 | 0 | 0 |
| 81 | STG.L | -53.16 | -20.68 | 7.13 | 0 | 11506 | 0 | 4058 | 88 | 0 | 1580 |
| 82 | STG.R | 58.15 | -21.78 | 6.8 | 0 | 14417 | 0 | 4201 | 0 | 39 | 3174 |
| 83 | TPOsup.L | -39.88 | 15.14 | -20.18 | 0 | 383 | 0 | 638 | 3974 | 14 | 1785 |
| 84 | TPOsup.R | 48.25 | 14.75 | -16.86 | 0 | 1660 | 0 | 543 | 3787 | 0 | 983 |
| 85 | MTG.L | -55.52 | -33.8 | -2.2 | 447 | 2858 | 4105 | 2133 | 45 | 903 | 22611 |
| 86 | MTG.R | 57.47 | -37.23 | -1.47 | 3531 | 1509 | 5639 | 2505 | 2 | 1954 | 15785 |
| 87 | TPOmid.L | -36.32 | 14.59 | -34.08 | 0 | 0 | 0 | 0 | 3257 | 0 | 1380 |
| 88 | TPOmid.R | 44.22 | 14.55 | -32.23 | 0 | 23 | 0 | 12 | 3959 | 0 | 2358 |
| 89 | ITG.L | -49.77 | -28.05 | -23.17 | 56 | 0 | 4466 | 0 | 6122 | 4149 | 6015 |
| 90 | ITG.R | 53.69 | -31.07 | -22.32 | 2847 | 0 | 7281 | 0 | 6634 | 4134 | 3882 |
