# Supplementary material for: Alteration of resting-state network dynamics in autism spectrum disorder based on leading eigenvector dynamics analysis
Source: Front Integr Neurosci. 2023 Jan 19;16:922577. doi: 10.3389/fnint.2022.922577 (PMC9892631; doi:10.3389/fnint.2022.922577)
Supplement: Supplementary file 2 [file Table_2.docx]

Table S2 The corresponding RSNs of the PL states (value = 1 represents the red nodes and the corresponding elements of centroid vectors is positive; value = 0 represents the blue red nodes and the corresponding elements is negative)

| **Index** | **Brain area** | **VAN-FPN-DMN** | **VIS** | **VIS-SMN-DMN** | **SUB-SMN-FPN** | **SUB-SMN-VAN** |
| --- | --- | --- | --- | --- | --- | --- |
| 1 | PreCG.L | 1 | 0 | 1 | 1 | 1 |
| 2 | PreCG.R | 0 | 0 | 0 | 1 | 1 |
| 3 | SFGdor.L | 1 | 0 | 0 | 0 | 0 |
| 4 | SFGdor.R | 1 | 0 | 1 | 1 | 0 |
| 5 | ORBsup.L | 1 | 0 | 0 | 0 | 0 |
| 6 | ORBsup.R | 1 | 0 | 0 | 0 | 0 |
| 7 | MFG.L | 1 | 0 | 1 | 1 | 0 |
| 8 | MFG.R | 1 | 1 | 1 | 1 | 0 |
| 9 | ORBmid.L | 1 | 0 | 0 | 1 | 0 |
| 10 | ORBmid.R | 1 | 0 | 0 | 1 | 0 |
| 11 | IFGoperc.L | 1 | 0 | 0 | 1 | 1 |
| 12 | IFGoperc.R | 1 | 0 | 0 | 1 | 1 |
| 13 | IFGtriang.L | 1 | 0 | 0 | 1 | 0 |
| 14 | IFGtriang.R | 1 | 0 | 0 | 1 | 0 |
| 15 | ORBinf.L | 1 | 0 | 0 | 0 | 0 |
| 16 | ORBinf.R | 1 | 0 | 0 | 0 | 1 |
| 17 | ROL.L | 0 | 0 | 0 | 1 | 1 |
| 18 | ROL.R | 0 | 0 | 0 | 1 | 1 |
| 19 | SMA.L | 1 | 0 | 1 | 1 | 1 |
| 20 | SMA.R | 1 | 0 | 1 | 1 | 1 |
| 21 | OLF.L | 0 | 0 | 0 | 0 | 0 |
| 22 | OLF.R | 0 | 0 | 0 | 0 | 0 |
| 23 | SFGmed.L | 1 | 0 | 0 | 0 | 0 |
| 24 | SFGmed.R | 1 | 0 | 0 | 0 | 0 |
| 25 | ORBsupmed.L | 0 | 0 | 0 | 0 | 0 |
| 26 | ORBsupmed.R | 0 | 0 | 0 | 0 | 0 |
| 27 | REC.L | 0 | 0 | 0 | 0 | 0 |
| 28 | REC.R | 0 | 0 | 0 | 0 | 0 |
| 29 | INS.L | 1 | 0 | 0 | 1 | 1 |
| 30 | INS.R | 1 | 0 | 0 | 1 | 1 |
| 31 | ACG.L | 1 | 0 | 0 | 0 | 0 |
| 32 | ACG.R | 1 | 0 | 0 | 0 | 0 |
| 33 | DCG.L | 1 | 0 | 1 | 1 | 0 |
| 34 | DCG.R | 1 | 0 | 1 | 1 | 0 |
| 35 | PCG.L | 0 | 0 | 1 | 0 | 0 |
| 36 | PCG.R | 0 | 1 | 1 | 0 | 0 |
| 37 | HIP.L | 0 | 0 | 0 | 0 | 1 |
| 38 | HIP.R | 0 | 0 | 0 | 0 | 1 |
| 39 | PHG.L | 0 | 0 | 0 | 0 | 1 |
| 40 | PHG.R | 0 | 0 | 0 | 0 | 1 |
| 41 | AMYG.L | 0 | 0 | 0 | 0 | 1 |
| 42 | AMYG.R | 0 | 0 | 0 | 0 | 1 |
| 43 | CAL.L | 0 | 1 | 1 | 0 | 0 |
| 44 | CAL.R | 0 | 1 | 1 | 0 | 0 |
| 45 | CUN.L | 0 | 1 | 1 | 0 | 0 |
| 46 | CUN.R | 0 | 1 | 1 | 0 | 0 |
| 47 | LING.L | 0 | 1 | 0 | 0 | 0 |
| 48 | LING.R | 0 | 1 | 1 | 0 | 0 |
| 49 | SOG.L | 0 | 1 | 1 | 0 | 0 |
| 50 | SOG.R | 0 | 1 | 1 | 0 | 0 |
| 51 | MOG.L | 0 | 1 | 1 | 0 | 0 |
| 52 | MOG.R | 0 | 1 | 1 | 0 | 0 |
| 53 | IOG.L | 0 | 1 | 0 | 0 | 0 |
| 54 | IOG.R | 0 | 1 | 0 | 0 | 0 |
| 55 | FFG.L | 0 | 1 | 0 | 0 | 1 |
| 56 | FFG.R | 0 | 1 | 0 | 0 | 1 |
| 57 | PoCG.L | 0 | 0 | 1 | 1 | 1 |
| 58 | PoCG.R | 0 | 0 | 1 | 1 | 1 |
| 59 | SPG.L | 0 | 1 | 1 | 1 | 0 |
| 60 | SPG.R | 0 | 1 | 1 | 1 | 0 |
| 61 | IPL.L | 1 | 1 | 1 | 1 | 0 |
| 62 | IPL.R | 1 | 1 | 1 | 1 | 0 |
| 63 | SMG.L | 1 | 0 | 1 | 1 | 1 |
| 64 | SMG.R | 1 | 0 | 1 | 1 | 1 |
| 65 | ANG.L | 1 | 0 | 1 | 0 | 0 |
| 66 | ANG.R | 1 | 1 | 1 | 0 | 0 |
| 67 | PCUN.L | 0 | 1 | 1 | 0 | 0 |
| 68 | PCUN.R | 0 | 1 | 1 | 0 | 0 |
| 69 | PCL.L | 0 | 0 | 1 | 1 | 1 |
| 70 | PCL.R | 0 | 1 | 1 | 1 | 0 |
| 71 | CAU.L | 1 | 0 | 0 | 0 | 0 |
| 72 | CAU.R | 1 | 0 | 0 | 1 | 0 |
| 73 | PUT.L | 0 | 0 | 0 | 1 | 1 |
| 74 | PUT.R | 0 | 0 | 0 | 1 | 1 |
| 75 | PAL.L | 0 | 0 | 0 | 1 | 1 |
| 76 | PAL.R | 0 | 0 | 0 | 1 | 1 |
| 77 | THA.L | 0 | 0 | 0 | 0 | 1 |
| 78 | THA.R | 0 | 0 | 0 | 1 | 1 |
| 79 | HES.L | 0 | 0 | 0 | 1 | 1 |
| 80 | HES.R | 0 | 0 | 0 | 1 | 1 |
| 81 | STG.L | 0 | 0 | 0 | 1 | 1 |
| 82 | STG.R | 0 | 0 | 0 | 1 | 1 |
| 83 | TPOsup.L | 1 | 0 | 0 | 0 | 1 |
| 84 | TPOsup.R | 1 | 0 | 0 | 1 | 1 |
| 85 | MTG.L | 0 | 0 | 0 | 0 | 1 |
| 86 | MTG.R | 0 | 0 | 0 | 0 | 0 |
| 87 | TPOmid.L | 0 | 0 | 0 | 0 | 1 |
| 88 | TPOmid.R | 0 | 0 | 0 | 0 | 1 |
| 89 | ITG.L | 0 | 0 | 0 | 0 | 1 |
| 90 | ITG.R | 0 | 1 | 0 | 0 | 0 |
